# The relationship between weight, eating behaviours and mental health over time in the YOUTH longitudinal cohort study

**DOI:** 10.1038/s41366-026-02058-7

**Published:** 2026-04-01

**Authors:** Megan Whatnall, Therese Fozard, Katerina Z. Kolokotroni, Tamla Evans, Jordan Marwood, Kim Colyvas, Louisa Ells, Tracy Burrows

**Affiliations:** 1 School of Health Sciences, College of Health, Medicine and Wellbeing, University of Newcastle, Newcastle, NSW Australia; 2 Food and Nutrition Program, Hunter Medical Research Institute, Newcastle, NSW Australia; 3 Centre for Psychological Research, School of Humanities and Social Sciences, Leeds Beckett University, Leeds, UK; 4 Medical Research Council Epidemiology Unit, School of Clinical Medicine, University of Cambridge, Cambridge, UK; 5 Obesity Institute, School of Health, Leeds Beckett University, Leeds, UK

**Keywords:** Risk factors, Weight management

## Abstract

**Background:**

Weight, eating behaviours and mental health have a complex interrelationship that is not fully understood. This study aimed to investigate the relationships between weight, eating behaviours and mental health over 12 months among socio-demographically diverse young adults (18–35 years) from the UK and Australia.

**Methods:**

Longitudinal analysis of data from the YOUTH cohort study was conducted. Three timepoints of data were used (baseline, 6 months, 12 months), collected between 2021–2023 using online surveys hosted via the Prolific platform. The dataset includes 507, 371 and 336 participants at the respective timepoints. Random-intercept cross-lagged panel models (RI-CLPM) were used to explore the relationships between eating behaviours (addictive, disordered and emotional eating) and mental health (stress, depression, anxiety, quality of life) with weight (kilograms) over the three timepoints.

**Results:**

Significant relationships were found between weight at baseline with Eating Disorder Examination Questionnaire (EDEQ) global score at 6 months (*β* = 0.028, *p* = 0.005, 95% CI = 0.009, 0.048); Positive-Negative Emotional Eating Scale (PNEES)—positive score at 6 months (*β* = −0.217, *p* = 0.011, 95% CI = −0.386, −0.053); and EDEQ shape concern score at 6 months (*β* = 0.040, *p* < 0.001, 95% CI = 0.019, 0.060) and 12 months (*β* = 0.034, SE = 0.010, *p* = 0.001, 95% CI = 0.014, 0.056). The relationships between EDEQ shape concern at baseline and weight at 12 months (*β* = 1.845, *p* = 0.005, 95% CI = −0.017, 2.927), and weight at baseline with quality of life at 12 months (*β* = −0.393, *p* = 0.034, 95% CI = −0.737, 0.031) were significant based on *p* value only. No other results were statistically significant for the other explanatory variables with weight.

**Conclusion:**

Longitudinal relationships were identified for higher weight with higher disordered eating, less eating in response to positive emotions and lower quality of life in this young adult cohort. Future research should include more longitudinal analyses of these relationships. Findings also support the need for screening of disordered eating and mental ill-health in young adults within weight management services.

## Introduction

Young adulthood (18–35 years) is an important life stage, given the changes that typically occur that can influence health behaviour and health status [1]. This includes changes in circumstances and responsibilities around employment, education and training, finances, living situation and social and family life. Secondly, health behaviours and conditions that are developed during this stage commonly track across adulthood, and influence short and long-term health outcomes [2–4]. Weight gain is greatest during young adulthood than in any other adult age group, and given the rise in weight-related comorbidities and deaths, such as cardiovascular diseases and diabetes, this is an important time to intervene and prevent weight gain [5, 6]. However, engagement and retention within existing weight management programmes are poor, with further research urgently required [7, 8].

Research into the relationships between weight and eating behaviours, specifically addictive, disordered and emotional eating behaviours, and weight and mental health, in young adults is expanding. Health states and behaviours are not static, and there is a lack of understanding of how these factors change and interrelate over time [9, 10]. This knowledge is critical in ensuring prevention and treatment services can effectively support positive change over time [11, 12]. In a nationally representative US cohort of young adults (*n* = 2785), food addiction was related to a 31% greater relative risk of weight control behaviours, and 50% greater relative risk of having overweight or obesity [13]. Further cross-sectional studies in young adults have found associations between higher cognitive restraint, higher emotional eating, and/or higher psychological distress with obesity measured in various ways, including body mass index (BMI) and body composition [12, 14–16]. In relation to mental health, a US cohort study of >1 million adolescents and young adults from 2017 to 2021 found that the incidence of depression increased more in those with obesity than those without [17]. Further, a 1-year longitudinal study of Brazilian university students (*n* = 583) during the COVID-19 pandemic found that in those living with overweight or obesity, previous moderate/severe anxiety symptoms were associated with increased consumption of ultra-processed foods at 12-month follow-up [18]. Meanwhile, studies from the UK and across Europe also identified worsening of lifestyle behaviours (e.g., diet, physical activity, sleep) and mental health (including depression and psychological distress) in those living with obesity [19, 20]. Studies also demonstrate differences in health behaviours and weight gain between genders. For example, higher likelihood of disordered eating among females [21], and higher risk of having overweight and obesity over time in young adult males than females [22]. However, as study samples are often predominantly female, this is another area in need of further enquiry, comparing relationships by gender in samples with adequate numbers of each.

Collectively, studies in young adulthood and general adulthood demonstrate that associations exist between increased weight and more problematic eating behaviours, and increased weight and higher symptomologies of stress and mental ill health, in young adults. However, studies are predominantly cross-sectional, and those that are longitudinal explore a limited set of measures under the constructs of eating behaviours and mental health with weight, and/or have not explored bi-directionally. Furthermore, many studies are in samples of university/college students who are largely, but not always entirely, in the young adult age range, and who are a subset of the young adult population that is not necessarily representative of the socioeconomic diversity of all young adults. For example, study samples tend to be predominantly female and highly educated. As such, gaps in the evidence base remain in terms of relationships over time and the direction of effects between weight, eating behaviours and mental health in young adults, which can only be explored using longitudinal data. Addressing these gaps will help further understand the relationship between mental health and weight status in young adults, and in turn inform the development of more tailored interventions. Further understanding of how the factors interrelate over time can inform more integrated care approaches that take into account potential multi-morbidities. The aim of the current study is therefore to investigate the relationships between weight, eating behaviours and mental health over a 12-month period among socio-demographically diverse young adults (18–35 years).

## Materials and methods

### Study design and participants

The YOUTH study is a longitudinal cohort study with an a priori protocol of three timepoints of data collection to date (baseline, 6 months and 12 months). Dates of data collection were: December 2021–February 2022, 6 months; June–July 2022, and 12 months; December 2022–January 2023. The current study addresses the pre specified objective of the data to investigate eating behaviours, mental health factors, personality, health-related behaviours and sociodemographics as contributors to weight change among young adults (18–35 years) over a 12-month period; To support longer-term evaluation and address identified research gaps, a fourth data collection time point has been added as = a post-trial follow up. This phase is currently underway (36 months; November 2024–current) and not included in the current paper. Data were collected via an online survey, hosted in the Qualtrics survey platform (Qualtrics LLC, Provo, Utah, USA), and distributed via the Prolific research platform (Prolific Academic Ltd., London, England). Prolific participants are paid for their time, as per platform standards. Participants received £5.00 per survey/timepoint. The protocol for the study is outlined in full in a previous publication [23]. Study conduct and reporting adhere to the Strengthening the Reporting of Observational Studies in Epidemiology (STROBE) guidelines for cohort studies [24].

Participant inclusion criteria were young adults aged 18–34 years at baseline, living in the UK or Australia, and with Body Mass Index (BMI) ≥ 20 kg/m^2^. Exclusion criteria were pregnant or trying to get pregnant, breastfeeding, or not fluent in the English language. A target of 500 participants was set, including 100 participants per BMI category, and 50:50 male:female and 75:25 UK/Australia within each category. The sample was stratified by BMI category and gender to ensure adequate numbers within each category for future analyses/to address under-representation in previous studies, whilst country was pragmatic based on the Prolific user base. Participants who met the inclusion criteria based on their demographics as collected by Prolific were invited to complete an eligibility screening questionnaire prior to joining the study. All participants who were eligible and completed the survey at baseline were invited to the 6-month and 12-month follow-up. Participants were still invited to complete the 12-month follow-up if they did not complete the 6-month follow-up.

### Study measures

The survey took approximately 45 min to complete at each timepoint, including questions within the following sections: demographics, weight management and health service usage, eating behaviours, personality, mental health, health-related behaviours, and others. Those measures included in this analysis are outlined below.

#### Sociodemographic characteristics

Sociodemographic characteristics included in this analysis are age, gender, country of residence, ethnic background, household income, highest level of education completed, and whether participants were enrolled at a university/college. Household income was compared with median national household income (including taxes and benefits) for UK/Australia in the year that the data were collected/nearest available, and is reported as ‘at or above’ or ‘below’ median national household income. In the UK, the median national household income was £38,200/year and £39,700/year (GBP) for the financial years ending 2022 and 2023, respectively [25]. In Australia, the nearest available data was from the 2021 Census, with a median national household income of $1770/week (AUD) [26].

#### Weight and height

Weight and height were self-reported. Participants reported their weight in either kilograms, pounds, or stone and pounds, and height in metres or feet and inches. Weight and height measures were converted into kilograms and metres, respectively, before calculating BMI using the standard equation (weight (kg)/height(m)^2^), and categorisation using World Health Organisation cut-points [27]. Self-reporting of weight in this study has been validated against images of weight captured on a set of scales [28].

#### Eating behaviours

The Modified Yale Food Addiction Scale 2.0 (mYFAS 2.0) was used to assess addictive eating [29]. This includes 13 questions relating to addictive eating behaviours and associated clinically significant distress and impairment. The number of addictive eating symptoms (0–11), categorisation of addictive eating (≥2 symptoms and endorsement of clinical distress or impairment) or not, and severity of addictive eating (2–3 symptoms = mild, 4–5 symptoms = moderate, ≥6 symptoms = severe) can then be scored. In this study, participants were asked to reflect on the past 6 months as opposed to the usual reference period of the past 12 months, so as to align with the timepoints of data collection.

The Eating Disorder Examination Questionnaire 6.0 (EDEQ) was used to assess for behavioural features of eating disorders [30]. The tool includes 22 questions relating to severity and six questions relating to frequency of key behavioural features, with a reference period of the past 28 days. The questions relating to severity are scored across four subscales (restraint, eating concern, shape concern, and weight concern) and an overall or global score, each with a score range of 0–6. Global scores of ≥3 were considered as ‘clinical range’ [31]. The tool has demonstrated validity, including the global and subscale scores, in several studies [32, 33]. Note that in our protocol paper, we specified a cut-off of ≥4 to be considered clinical range [23], however this has been adjusted on the basis that more recent studies indicate this to be too high [34], and that a cut-off of ≥3 is used in the Australian healthcare system to determine eligibility for eating disorder services [31].

The Positive-Negative Emotional Eating Scale was used to assess emotional eating [35]. This tool includes seven questions relating to positive emotional eating and 12 relating to negative emotional eating, which are scored from 0/Never to 4/Very often. Subscale scores range from 0–28 and 0–48 for the positive and negative subscales, respectively, with higher scores relating to a higher likelihood of eating in response to positive/negative emotions.

#### Mental health

The 4-item Perceived Stress Scale 4 (PSS 4) was used to assess stress [36]. This questionnaire assesses stress in daily life over the past month from 0/Never to 4/Very often, with a total score then calculated from 0–16. A higher score indicates higher perceived stress.

The Centre for Epidemiological Studies Depression Scale 10-item questionnaire (CES-D-10) was used to assess for depression [37]. The questionnaire assesses the frequency of depressive symptoms in the past week, from 0/Less than 1 day to 3/5–7 days, with a total score then calculated from 0–30. A higher score indicates greater depressive symptoms.

The Generalised Anxiety Disorder 7-item Scale (GAD-7) was used to assess for anxiety [38]. The questionnaire assesses the frequency of anxiety symptoms in the past 2 weeks from 0/Not at all to 3/Nearly every day, with a total score then calculated from 0–21. A higher score indicates higher anxiety, while scores can also be categorised into minimal (0–4), mild (5–9), moderate (10–14) or severe (15–21) anxiety.

The EQ-5D was used to assess health-related quality of life [39]. This is a 6-item tool assessing health across the domains of mobility, self-care, usual activities, pain/discomfort, and anxiety/ depression, as well as assessing overall health ranked on a scale of 0/Worst health imaginable to 100/ Best health imaginable. Only the ranking of the overall health item was included in the current analysis.

### Statistical analysis

Descriptive statistics are reported as median and inter-quartile range for continuous data, as data had skewed distributions, and numbers and percentages for categorical data. Differences in participant demographic, weight, eating behaviour and mental health characteristics at baseline were assessed between those who completed baseline only and those who completed one or both follow-up surveys using ANOVA or chi-square tests.

Random-intercept cross-lagged panel models (RI-CLPM) [40] were used to explore the relationships between eating behaviour and mental health measures with weight over the three timepoints. The weight variable used in analyses was weight in kilograms. Preliminary CLPM modelling, i.e., without the random intercepts, indicated that both auto-regressive paths and cross-lagged paths between times 1 and 2 and time 2 and 3 did not differ. As a result, these paths were constrained to be the same for the RI-CLPM modelling. The modelled relationships are outlined in the Supplementary Materials (Supplementary Fig. 1) along with a description to assist in understanding model components. The models were fit with the estimator option MLR, which is suitable for missing data, performing a maximum likelihood estimation and outputting robust standard errors. To assess model fit, robust versions of the comparative fit index (CFI), root mean square error of approximation (RMSEA), Standardized Root Mean Square Residual (SRMR) and Tucker-Lewis Index (TLI) were used. The following benchmarks were used to indicate a good fit of the model to the data: CFI >0.95, RMSEA ≤ 0.05, SRMR ≤ 0.05 and TLI >0.95 [41, 42]. Statistical significance was set at the 0.05 level. Calculated parameters for long-run effects [43] spanning times 1 to 3 were examined as well as single-step regression coefficients for times 1 to 2 or 2 to 3. Monte Carlo simulation based 95% CIs were determined for all effects. An estimation issue for the weight random effect parameter was overcome by fixing the variance for this term at 790, the value before the autoregressive terms for weight were entered into the model. If this wasn’t done, the variance for the random effect became negative, and all the substantial between-participant weight variability was transferred to the time 1 weight variance, breaking the model’s correct representation of the sources of variability in the system. As the study was exploratory in nature, we chose not to adjust statistical significance for the multiple explanatory variables examined.

Multiple group models were also computed to test for differences by gender. Only participants identifying as male and female at baseline (*n* = 501) were included in these models, as the number of participants identifying as non-binary or another gender identity was too small to enable comparison. The fixing of the variance for the weight random effect was not carried out for the gender comparisons. Robust chi-square difference test statistics were used to determine if there was a significant difference between genders [44].

As explained in [44] the RI-CLPM was partitioned into between and within person variation. The between-component, capturing trait-like variation that was stable over time. The within-person variation reflecting individuals’ shorter-term differences around their longer-term trait scores were the focus of the analysis. Two types of regression relationships were obtained from the model. The first estimating auto-regressive effects that account for temporal stability, “the amount of within-person carryover effect”, These effects were of secondary interest (not reported), their primary purpose to ensure that the cross-lagged paths were estimated without bias due to these carry-over effects. These RI terms and autoregressive effects were also estimated for all of the eating behaviour or mental health measures in a set of models where weight was paired in turn with each of these variables. The remaining unexplained variability in each model pair was where the relationships of most interest were obtained, the cross-lagged terms. The size of the regression coefficients for these reflects the size of (Granger) causal relationships between the variables. As an illustration of a cross-lagged term, weight at the first time period might have a causal effect on an eating disorder or mental health variable at time 2. It is these coefficients that are presented in Table 1. They are unstandardised cross-lagged regression coefficients for times 1 to 2, long-run path parameters for the cross-lagged estimates (times 1 to 3) and also model fit indices. Note that the cross-lagged estimates from times 2 to 3 were fixed in the modelling, so these path estimates are the same as those for times 1 to 2. Model fit indices indicate a good fit to the data for all models with the exception of the models of weight with EDEQ restraint, EDEQ eating concern, EDEQ weight concern, stress, and anxiety, where the RMSEA were >0.05.

**Table 1:** Summary of RI-CLPM outcomes: unstandardised cross-lagged path regression coefficients and long-run path parameters (standard error) for the YOUTH cohort study (*n* = 507).

Weight & x	Unstandardised regression coefficients or long run path parameters (times T1 to T3)^a^				Model fit indices			
	x at T1 to weight at T2	x at T1 to weight at T3	Weight at T1 to x at T2	Weight at T1 to x at T3	CFI	RMSEA	SRMR	TLI
Weight & mYFAS 2.0	0.780 (0.562)	0.393 (0.634)	0.026 (0.062)	0.013 (0.043)	0.998	0.050	0.025	0.995
Weight & EDEQ Global	2.189 (3.027)	1.458 (1.761)	0.028 (0.010)	0.019 (0.042)	0.998	0.050	0.038	0.996
Weight & EDEQ Restraint	0.367 (0.797)	0.083 (0.352)	0.028 (0.024)	0.006 (0.019)	0.996	0.066	0.030	0.991
Weight & EDEQ Eating concern	1.256 (0.708)	0.518 (0.435)	0.015 (0.011)	0.006 (0.007)	0.995	0.078	0.032	0.989
Weight & EDEQ Shape concern	2.127 (1.103)	1.845 (0.655)*	0.040 (0.010)	0.034 (0.010)	0.998	0.050	0.045	0.996
Weight & EDEQ Weight concern	0.634 (1.788)	0.345 (1.172)	0.012 (0.042)	0.007 (0.027)	0.998	0.052	0.024	0.995
Weight & PNEES negative	0.027 (0.111)	0.023 (0.092)	0.083 (0.163)	0.072 (0.124)	1.000	0.000	0.026	1.001
Weight & PNEES positive	−0.174 (0.190)	−0.070 (0.105)	−0.217 (0.085)	−0.087 (0.071)	0.998	0.047	0.020	0.995
Weight & Stress	0.224 (0.372)	0.060 (0.138)	0.010 (0.027)	0.003 (0.009)	0.997	0.055	0.022	0.993
Weight & Anxiety	0.141 (0.238)	0.027 (0.081)	−0.000 (0.103)	−0.000 (0.020)	0.997	0.058	0.023	0.993
Weight & Depression	0.157 (0.117)	0.189 (0.145)	0.069 (0.039)	0.083 (0.043)	1.000	0.022	0.021	0.999
Weight & QOL	−0.015 (0.031)	−0.016 (0.035)	−0.363 (0.198)	−0.393 (0.186)*	1.000	0.000	0.023	1.001

The weight variable used in analyses was weight in kilograms. Significant (*p* < 0.05), *not significant based on confidence interval.

*CFI* comparative fit index, *RMSEA* root mean square error of approximation, *SRMR* Standardized Root Mean Square Residual, *TLI* Tucker-Lewis Index.

^a^y = weight, x = explanatory variable as listed in the first column, the index is time period, 1, 2 or 3.

Descriptive statistics were generated using Stata software version 14.2 (StataCorp LLC, College Station, Texas, USA). RI-CLPM analyses were conducted using R statistical software V 4.3.1 (R Core Team (2021). Vienna, Austria), using the packages lavaan version 0.6-18 [45] and semTools [46].

## Results

### Summary of cohort characteristics

**Fig. 1:**
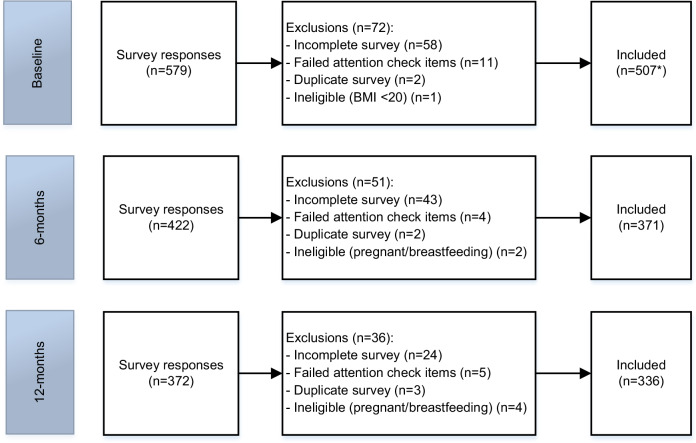
Flow diagram of participants in the YOUTH cohort study. *N = 508 included in previous publication using baseline data [28]. *N* = 1 later excluded due to failing attention check items in follow-up surveys.

The cohort included 507 at baseline, 371 at 6-month follow-up and 336 at 12-month follow-up. This equates to retention rates of 73% and 66% at 6 and 12-month follow-up, respectively. Figure 1 provides full details on participant numbers at each stage. The median age of the cohort at baseline was 29 years (IQR 24–32 years), with 49.5% females, 82.1% living in the UK, 78.1% of white ethnicity, and 55.2% with a household income at or above the median national income (Table 2). There were no significant differences in the demographic characteristics of participants at baseline between those who completed the baseline survey only and those who completed one or both follow-up surveys.

**Table 2:** Demographic characteristics of the YOUTH cohort study participants by timepoint.

	Baseline (*n* = 507)	6 months (*n* = 371)	12 months (*n* = 336)
Age (years), median (interquartile range)	29 (24–32)	29 (25–32)	30 (26–33)
Weight (kg), median (interquartile range)	96.1 (78.1–115.0)	96.2 (78.5–114.3)	98.0 (78.7–119.1)
BMI (kg/m^2^), median (interquartile range)	33.2 (27.0–39.4)	32.8 (26.6–37.9)	33.2 (27.2–38.6)
BMI category (kg/m^2^), *N* (%)			
<18	–	1 (0.3)	1 (0.3)
18–24.9	93 (18.3)	68 (18.3)	59 (17.6)
25–29.9	98 (19.3)	84 (22.6)	69 (20.5)
30–34.9	106 (20.9)	70 (18.9)	65 (19.4)
35–39.9	89 (17.5)	78 (21.0)	70 (20.8)
≥40	121 (23.8)	70 (18.9)	72 (21.4)
Gender, *N* (%)			
Female	251 (49.5)	190 (51.2)	162 (48.2)
Male	250 (49.3)	176 (47.4)	169 (50.3)
Non-binary	6 (1.2)	4 (1.1)	4 (1.2)
Another gender identity	–	1 (0.3)	1 (0.3)
Country, *N* (%)			
UK	416 (82.1)	312 (84.1)	282 (83.9)
Australia	91 (17.9)	59 (15.9)	54 (16.1)
Highest level of education, *N* (%)			
No formal qualifications	5 (1.0)	2 (0.5)	–
General Education Development tests (GED), General Certificate of Secondary Education (GCSE), School Certificate (or equivalent)	46 (9.1)	30 (8.1)	29 (8.6)
High school diploma, A-levels, or Higher School Certificate (or equivalent)	121 (23.9)	83 (22.4)	72 (21.4)
Technical/Community college	55 (10.9)	42 (11.3)	43 (12.8)
University degree (undergraduate)	191 (37.7)	142 (38.3)	126 (37.5)
Higher university degree (PhD, Master's, Graduate Diploma)	89 (17.6)	72 (19.4)	66 (19.6)
Current university/college student, *N* (%)	123 (24.3)	85 (22.9)	67 (19.9)
Household income, *N* (%)			
At or above the median national income^a^	233 (46.0)	182 (49.1)	166 (49.4)
Below the median national income	251 (49.5)	170 (45.8)	154 (45.8)
Unsure/chose not to disclose	23 (4.5)	19 (5.1)	16 (4.8)
Ethnic group, *N* (%)			
White	396 (78.1)	292 (78.7)	263 (78.3)
South Asian	36 (7.1)	29 (7.8)	24 (7.1)
Other Asian background	20 (3.9)	11 (3.0)	11 (3.3)
Black/African/Caribbean/Black British	18 (3.6)	14 (3.8)	14 (4.2)
Aboriginal or Torres Strait Islander	4 (0.8)	1 (0.3)	1 (0.3)
Mixed/multiple ethnicity groups	27 (5.3)	20 (5.4)	18 (5.4)
Other ethnicity group/s	6 (1.2)	4 (1.1)	5 (1.5)

^a^Median national household income in the UK (financial year ending 2022 = £38,200/year and 2023 = £39,700/year) and Australia (2021 Census = $1770/week).

The proportion of the cohort reporting addictive eating at baseline was 9.9%, while 33.5% reported disordered eating symptoms at or above clinical range, and 20.9% were categorised as living with severe anxiety (Table 3). The median anxiety score was seven out of a maximum 21, the median stress score was eight out of a maximum 16, the median depression score was 12 out of a maximum 30, and the median rating of overall health was 70 out of a maximum 100. There were no significant differences in the eating behaviours and mental health characteristics of participants at baseline between those who completed the baseline survey only and those who completed one or both follow-ups. Eating behaviour and mental health characteristics are also presented by BMI category and timepoint in Table 4, and Supplementary Fig. 2 presents this data visually.

**Table 3:** Eating behaviour and mental health characteristics of the YOUTH cohort study participants by timepoint.

	Baseline (*n* = 507)	6 months (*n* = 371)	12 months (*n* = 336)
Addictive eating (mYFAS)			
- Symptom score (/11)	1 (0–4)	1 (0–4)	1 (0–3)
- Addictive eating, *n* (%)	50 (9.9)	28 (7.5)	26 (7.7)
Disordered eating (EDE-Q)			
- Global score (/6)	2.4 (1.4–3.3)	2.4 (1.3–3.4)	2.3 (1.2–3.2)
- Restraint subscale (/6)	1.6 (0.6–2.8)	1.6 (0.4–2.6)	1.2 (0.4–2.6)
- Eating concerns subscale (/6)	1.2 (0.4–2.2)	1.2 (0.4–2.4)	1.0 (0.2–2.1)
- Shape concerns subscale (/6)	3.6 (2.1–4.6)	3.5 (2.1–4.6)	3.3 (1.8–4.6)
- Weight concerns subscale (/6)	3.2 (1.6–4.0)	3.2 (1.6–4.2)	2.8 (1.4–4.0)
- Clinical range (global score ≥3), *n* (%)	170 (33.5)	124 (33.4)	98 (29.2)
Emotional eating (PNEES)			
- Positive subscale (/48)	7 (3–11)	6 (2–11)	6 (2–11)
- Negative subscale (/28)	24 (11–34)	24 (12–35)	22 (8–33)
Stress (PSS-4) (/16)	8 (7–9)	8 (7–9)	8 (7–9)
Depression (CES-D-10) (/30)	12 (9–16)	11 (8–16)	12 (7–16)
Anxiety (GAD-7) (/21)	7 (4–13)	7 (4–12)	7 (3–12)
- Severe anxiety, *n* (%)	106 (20.9)	60 (16.2)	56 (16.7)
Health-related quality of life (EQ-5D)			
- Overall health rating (0–100)	70 (56–80)	71 (59–82)	71 (60–83)

Values are presented as median (interquartile range) unless otherwise noted.

**Table 4:** Eating behaviour and mental health characteristics of the YOUTH cohort study participants by BMI category and timepoint.

BMI category	Baseline (*n* = 507)	6 months (*n* = 371)	12 months (*n* = 336)
Addictive eating (mYFAS) (/11)			
18–24.9 kg/m^2^	0 (0–2)	0 (0–1)	0 (0–1)
25–29.9 kg/m^2^	0 (0–3)	0 (0–2)	0 (0–2)
30–34.9 kg/m^2^	1 (0–5)	1 (0–4)	1 (0–4)
35–39.9 kg/m^2^	3 (1–5)	3 (0–6)	2.5 (0–5)
≥40 kg/m^2^	3 (1–5)	3 (1–5)	2 (0–4)
Disordered eating (EDE-Q global) (/6)			
18–24.9 kg/m^2^	1.1 (0.5–2.0)	1.0 (0.4–2.1)	0.6 (0.2–1.5)
25–29.9 kg/m^2^	2.1 (1.2–3.2)	2.1 (1.0–2.9)	1.9 (1.1–3.1)
30–34.9 kg/m^2^	2.3 (1.7–3.3)	2.5 (1.3–3.6)	2.5 (1.4–3.3)
35–39.9 kg/m^2^	2.9 (2.2–3.7)	2.9 (2.1–3.7)	2.6 (1.9–3.4)
≥40 kg/m^2^	2.8 (2.2–3.5)	3.1 (2.3–3.9)	2.8 (2.1–3.7)
Emotional eating (PNEES)—Positive subscale (/28)			
18–24.9 kg/m^2^	6 (1–13)	6 (2–12)	4 (2–13)
25–29.9 kg/m^2^	7 (2–10)	5 (2–10)	7 (2–10)
30–34.9 kg/m^2^	7 (3–10)	7 (2–11)	6 (2–10)
35–39.9 kg/m^2^	8 (5–13)	7 (3–12)	6.5 (2–11)
≥40 kg/m^2^	7 (3–10)	7 (4–12)	7 (3–11)
Emotional eating (PNEES)—Negative subscale (/48)			
18–24.9 kg/m^2^	13 (3–26)	12.5 (1–20.5)	8 (2–22)
25–29.9 kg/m^2^	21 (12–31)	16.5 (9–26)	18 (6–29)
30–34.9 kg/m^2^	22.5 (12–33)	24 (12–36)	17 (11–30)
35–39.9 kg/m^2^	32 (17–37)	29.5 (20–37)	25.5 (14–36)
≥40 kg/m^2^	27 (16–37)	31.5 (24–40)	31 (21.5–39)
Stress (PSS-4) (/16)			
18–24.9 kg/m^2^	8 (7–9)	8 (7–8)	8 (7–9)
25–29.9 kg/m^2^	8 (7–9)	8 (7–9)	8 (7–9)
30–34.9 kg/m^2^	8 (7–9)	8 (7–9)	8 (7–8)
35–39.9 kg/m^2^	8 (7–10)	8 (7–9)	8 (7–9)
≥40 kg/m^2^	8 (7–9)	8 (7–9)	8 (7–9)
Depression (CES-D-10) (/30)			
18–24.9 kg/m^2^	10 (8–14)	9 (6.5–13)	8 (6–12)
25–29.9 kg/m^2^	12 (9–15)	10 (7–14.5)	10 (7–14)
30–34.9 kg/m^2^	11 (9–16)	12 (8–16)	12 (8–16)
35–39.9 kg/m^2^	12 (10–16)	13 (9–17)	12.5 (9–18)
≥40 kg/m^2^	13 (10–17)	14 (9–18)	15 (10–18)
Anxiety (GAD-7) (/21)			
18–24.9 kg/m^2^	6 (2–12)	5 (2–8.5)	5 (2–9)
25–29.9 kg/m^2^	7 (4–11)	7 (3–10)	6 (3–10)
30–34.9 kg/m^2^	8.5 (5–15)	7 (5–12)	7 (3–11)
35–39.9 kg/m^2^	9 (5–15)	9 (5–15)	8 (5–14)
≥40 kg/m^2^	9 (6–14)	10 (5–14)	10 (5.5–15.5)
Health related quality of life (EQ-5D) (/100)			
18–24.9 kg/m^2^	80 (70–87)	80 (70–90)	80 (70–90)
25–29.9 kg/m^2^	74 (61–81)	75 (63–82)	78 (66–85)
30–34.9 kg/m^2^	70 (55–80)	70 (55–83)	69 (60–80)
35–39.9 kg/m^2^	67 (54–79)	65 (50–80)	70 (56–80)
≥40 kg/m^2^	63 (40–75)	65 (41–76)	64 (40–79.5)

Values are presented as median (interquartile range). BMI category <18 kg/m^2^ not reported due to low numbers (*N* = 0 at baseline, *N* = 1 at 6 and 12 months).

### Random-intercept cross-lagged panel model (RI-CLPM) results exploring the relationships between eating behaviour and mental health measures with weight over time

The relationships between weight at baseline with EDEQ global at 6 months (*β* = 0.028, SE = 0.010, *p* = 0.005, 95% CI = 0.009, 0.048), and weight at baseline with PNEES positive at 6 months (*β* = −0.217, SE = 0.085, *p* = 0.011, 95% CI = −0.386, −0.053) were found to be significant and positive. Significant positive relationships were also found between weight at baseline and EDEQ shape concern at 6 months (*β* = 0.040, SE = 0.010, *p* < 0.001, 95% CI = 0.019, 0.060) and at 12 months (*β* = 0.034, SE = 0.010, *p* = 0.001, 95% CI = 0.014, 0.056). The relationship between EDEQ shape concern at baseline and weight at 12 months was positive and significant based on *p* value, but not significant based on confidence intervals (*β* = 1.845, SE = 0.655, *p* = 0.005, 95% CI = −0.017, 2.927). The relationship between weight at baseline with quality of life at 12 months was negative and significant based on *p* value, but not significant based on confidence intervals (*β* = −0.393, SE = 0.186, *p* = 0.034, 95% CI = −0.737, 0.031). This inconsistency between these two tests likely due to a lack of normality in the simulation based distribution of the 95% CI whereas the model based significance test assumes normality. The 95% CI bases significance is more likely to be correct. No other statistically significant results were found for other explanatory variables with weight. Within-person cross-lagged effects indicated that when individuals’ weight at baseline was higher than their own average across time, their EDEQ and PNEES-positive scores 6 months later were also higher than their usual levels, suggesting that short-term increases in weight were associated with subsequent lower than usual levels of eating disorder symptoms and positive emotional eating.

### RI-CLPM results exploring the relationships between eating behaviour and mental health measures with weight over time, by gender

No significant differences were found in the relationships between weight with the eating behaviour and mental health explanatory variables by gender. Model fit indices indicate a good fit to the data for all models. Results of the RI-CLPM analyses by gender are presented in Supplementary Table 1 and corresponding model fit indices in Supplementary Table 2.

## Discussion

This study investigated the bi-directional relationships between weight and eating behaviours, and weight and mental health, over a 12-month period in young adults. Significant relationships over time were found for weight with disordered eating, eating in response to positive emotions, and quality of life, while relationships between weight and addictive eating, eating in response to negative emotions, stress, anxiety and depression over time were not found to be significant. No significant differences were identified in these relationships between men and women. Although statistically significant findings were few, there were interesting patterns over time which were enabled with the multiple time points assessed over a 12 month period. As most studies exploring these relationships are cross-sectional, the current study is an important addition to the evidence base.

The current study found that weight at baseline was significantly related to disordered eating at both the 6 and 12-month follow-ups, assessed using the EDEQ. Specifically, higher weight at baseline was related to higher EDEQ global score at 6 months, as well as higher EDEQ shape concern subscale at both 6 and 12 months. This finding agrees with previous recent research that suggest that those of higher body weight who may or may not be seeking support from health professionals should have concurrent measures of disordered eating assessed during treatment, as these increasingly co-occur. A range of EDs and DE can co-occur in people of with higher weight, with BED the most prevalent among adults seeking obesity treatment. A recent meta-analysis found substantially higher rates of DE and EDs among adults seeking obesity treatment, with 14% meeting diagnostic criteria via clinical interviews and 26% reporting moderate binge eating using self-reported measures, compared to 3% of adults in the community [47]. Additionally, cross-sectional studies in women aged 16–50 years have found associations between higher BMI and higher EDEQ global scores [48, 49], as well as higher shape and weight concern sub-scores in individuals with higher versus lower BMI. The current analysis suggests that this relationship extends over time. While the relationship between EDEQ shape concern at baseline was also significantly positively related to weight at 12 months in the current study, this was significant based on p value and not confidence interval. It therefore seems that higher weight is more indicative of future disordered eating symptoms as opposed to the other way around.

Weight was also found to be significantly related to eating in response to positive emotions in the current study, measured using the PNEES. In this instance the relationship was negative, indicating that higher weight at baseline was related to a lower likelihood of eating in response to positive emotions over time. The relationship of weight with the PNEES negative subscale, i.e. eating in response to negative emotions, was not found to be significant in either direction. A 2023 review on the association of emotional eating with overweight/obesity concluded that an association does exist, whereby individuals with overweight or obesity report higher emotional eating [50]. One study in the review considered positive and negative aspects of emotional eating within a latent profile analysis of >600 adult women, finding four profiles, individuals with obesity and high positive/high negative emotional eating; individuals with BMI 18.5–24.9 kg/m^2^ and low positive/low negative emotional eating; individuals with overweight and low-moderate positive/moderate negative emotional eating; and individuals with BMI 18.5–24.9 kg/m^2^ and high negative/moderate-high positive emotional eating [51]. Showing some consistency with the current study results, no profile emerged where individuals scored highly on only negative emotional eating. Other contributing factors for the results found include age-specific behavioural patterns, characteristics of the PNEES measure, or the possibility that emotional eating co-occurs with but does not predict changes in weight. The time lag of measures in the YOUTH study (i.e., 6-monthly) and sample could also have contributed to this finding. Collectively, the evidence suggests that there is a complexity to emotional eating in relation to weight that requires further enquiry.

The current study also found that higher weight at baseline was significantly related to lower health related quality of life at 12 month follow up. This finding is consistent with the Yorkshire Health Study of 64,631 adults (>16 years), which found increasing levels of obesity to be associated with lower health-related quality of life [52]. The difference was greater between individuals with obesity compared with BMI of 18.5–24.9 kg/m^2^, than those with overweight compared with BMI of 18.5–24.9 kg/m^2^. Although differences were not statistically significant, the median health related quality of life across BMI groups in the current study aligns with this. At baseline, median QOL score was 63/100 in the obesity class III group compared with 74/100 and 80/100 in the overweight and 18.5–24.9 kg/m^2^ groups, respectively.

It was expected that more relationships would have been statistically significant between the measures of interest in this study, based on the evidence from cross-sectional studies and the limited evidence currently available from longitudinal studies. In terms of mental health and weight for example, longitudinal analysis of the associations between mood disorders and anxiety with BMI change in an Australian cohort of young adults found that history of mood disorders at baseline was associated with BMI gain at the 5-year follow up in males but not females, while the reverse was not significantly associated for either gender [53]. BMI and anxiety were not found to be significantly associated for either gender, in either direction. Further, a study of college students in the USA found that higher stress levels were associated with greater weight gain after one semester of college among male but not female students [54]. Systematic review evidence has also demonstrated the association between higher addictive eating and higher BMI [55]. In contrast, the current study found minimal significant relationships between the parameters of interest, including no differences between genders. It could be that a larger sample size is needed to power certain analyses, or that different time periods of follow up might identify significant differences. This is worth noting, the youth cohort at the group level included a moderate sample size (based on financial limitations), but when categorised by BMI or sex, the numbers of participants within each group significantly reduces.

While it is acknowledged that a fourth wave of data collection is underway, the current analysis included outcomes collected over three timepoints with 6-monthly intervals from baseline. The time use of survey measures may have contributed to insignificant results. For example, the timeframe between collection points may have been too short to detect changes or trends in some behavioural outcomes. While for other outcomes, the timeframe may have been too long to detect changes. Due to the self-report nature of the survey measures, findings may be susceptible to social desirability bias. The current study was not able to capture some information that may have also masked findings. For example, it is unknown whether study participants were actively seeking treatment for or trying to manage their weight, eating behaviours and mental health during the study period. Further, Covid was still prevalent during the data collection period which could also have impacted mental health and weight outcomes. There are range of analysis approaches for longitudinal data and the approach undertaken in the current study was guided by an expert statistician. However, the approach taken may have introduced some bias. Notwithstanding these potential limitations, this study provides an important guide for future research, including analyses in the YOUTH Cohort data across the breadth of other measures assessed (e.g. personality, health-related behaviours, other sociodemographic characteristics) to explore differences and/or moderating/mediating relationships between variables.

The equal proportion of female and male participants in the cohort, as well as the equal proportion of participants across BMI category are particular strengths, as they allow for a more accurate assessment of the research question within each of these groups. The small numbers within some other characteristics, including ethnicity, is a limitation in terms of analysing for differences based on these factors. High retention rates were achieved in this study overall and across participants from different demographics, especially considering the longitudinal aspect and the population group of young adults [7]. A further strength and novelty is the use of the Prolific platform for a study of this duration, of which there are few with follow up beyond 6 months [56]. In terms of the analysis, robust statistical methods were employed, giving strength to the reliability of the reported results.

In conclusion, this longitudinal analysis found evidence for relationships between higher weight with higher disordered eating scores, lower scores for eating in response to positive emotions and lower quality of life in a cohort of young adult men and women. There was a lack of significant findings between weight and eating in response to negative emotions, stress, anxiety and depression, as well as no differences between genders. Future research should include more longitudinal analyses of these relationships, considering additional factors such as other health and demographic factors. In terms of implications for practice, the study findings support the need for screening of disordered eating and mental ill-health in young adults within weight management services.

## Supplementary information

Supplementary Material

## Data Availability

Data described in the manuscript may be made available upon request pending application and approval.
