# Supplementary material for: The relationship between weight, eating behaviours and mental health over time in the YOUTH longitudinal cohort study
Source: Int J Obes (Lond). 2026 Apr 1;50(6):1258–66. doi: 10.1038/s41366-026-02058-7 (PMC13286977; doi:10.1038/s41366-026-02058-7)
Supplement: Supplementary file 1 — Supplementary Material [file 41366_2026_2058_MOESM1_ESM.docx]

**Supplementary Figure 1: Random Intercept Cross-Lagged Panel Model (RI-CLPM) with Three Time Points of the YOUTH cohort**


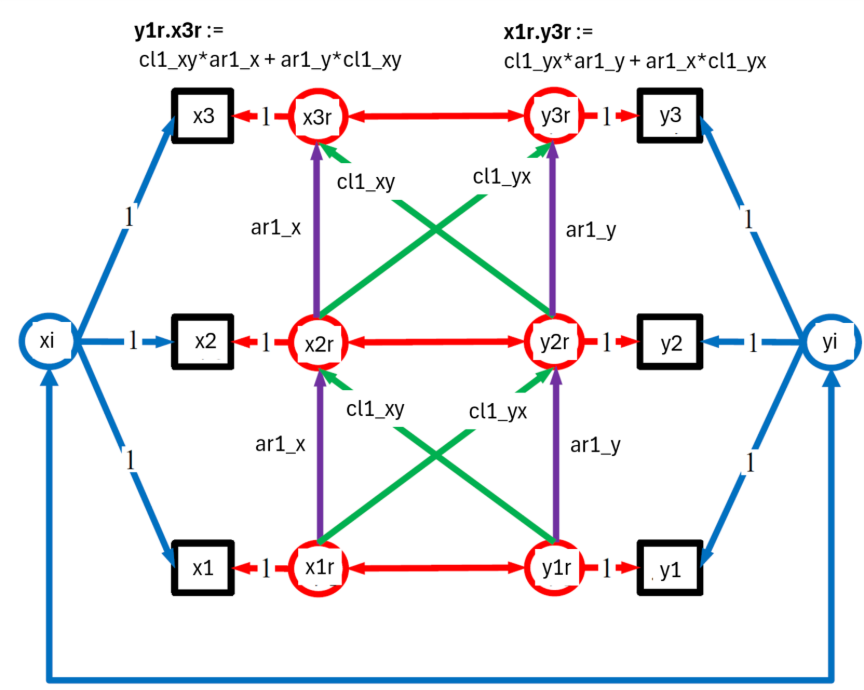


This diagram illustrates the random intercept cross-lagged panel model (RI-CLPM) for a pair of variables X and Y with three time points (x1 or y1 baseline, x2 or y2 6 months, and x3 or y3 for 12 months). The random intercepts are the xi and yi variables with paths fixed to 1 for the three observed X variables, x1,x2 and x3 and three observed Y variables, y1,y2 and y3. By adjusting for the random intercepts for each person on both the X and Y variables residuals were formed indicated by the x or y variables with suffix r, x1r, y1r, …, x3r,  y3r. The regression relationships in the RI-CLPM were based on these residuals.

For this study Y was always weight and the X variable was in turn one of the eating disorder or mental health variables. Two types of regression paths were estimated. the autoregressive paths (in purple) within each of the variables, e.g. x1r to x2r or y1r to y2r where the regression coefficients were labelled ar1_x or ar1_y. and the cross-lagged paths (in green), e..g y1r to x2r.

Note a parsimonious model was chosen where the autoregressive paths were chosen to be equal, i.e. x1r to x2r = x2r to x3r = ar1_x and similarly for the y residuals, set equal to ar1_y. Also the cross-lagged paths were constrained to be equal, x1r to y2r = x2r to y3r = cl1_yx and similarly for the cross-lagged paths originating from y1r and y2r = cl1_xy. This decision was based on model comparison tests with the simpler CLPM.

The longer 2 step paths were estimated according to the 2 formulae above the figure. For example. the effect of a disordered eating or mental health variable at time 1 on weight at time 3 the formula was estimated using

x1r.y3r = cl1_yx*ar1_y + ar1_x*cl1_yx

This formula can be understood using the RI-CLPM figure above and following the paths identified by the labels in the formula. Two paths are described (each of 2 steps) by which residuals (called impulses in [47]) at x1r contribute to effects at y3r.

The first of the 2 paths estimated using cl1_yx*ar1_y is for 2 steps,

1. firstly from x1r to y2r via the cross-lagged path cl1_yx and then
2. y2r to y3r via the autoregressive path ar1_y (noting that this path had been constrained to be equal to the autoregressive path y1r to y2r).

The second way that effects from x1r can be transmitted to y3r are by an alternative pathway,

1. from x1r to x2r via the autoregressive path ar1_x and then
2. x2r to y3r via the cross-lagged path cl1_yx ((noting that this path had been constrained to be equal to the cross-lagged path x1r to y2r)

The effect then of x1r on y3r is then sum of the two, 2 step path components.


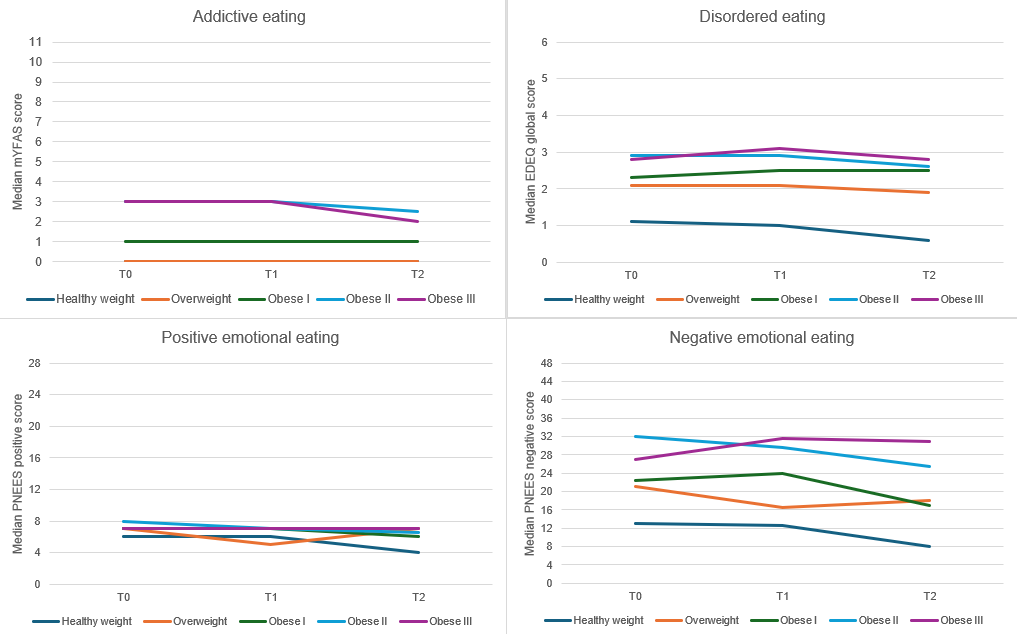


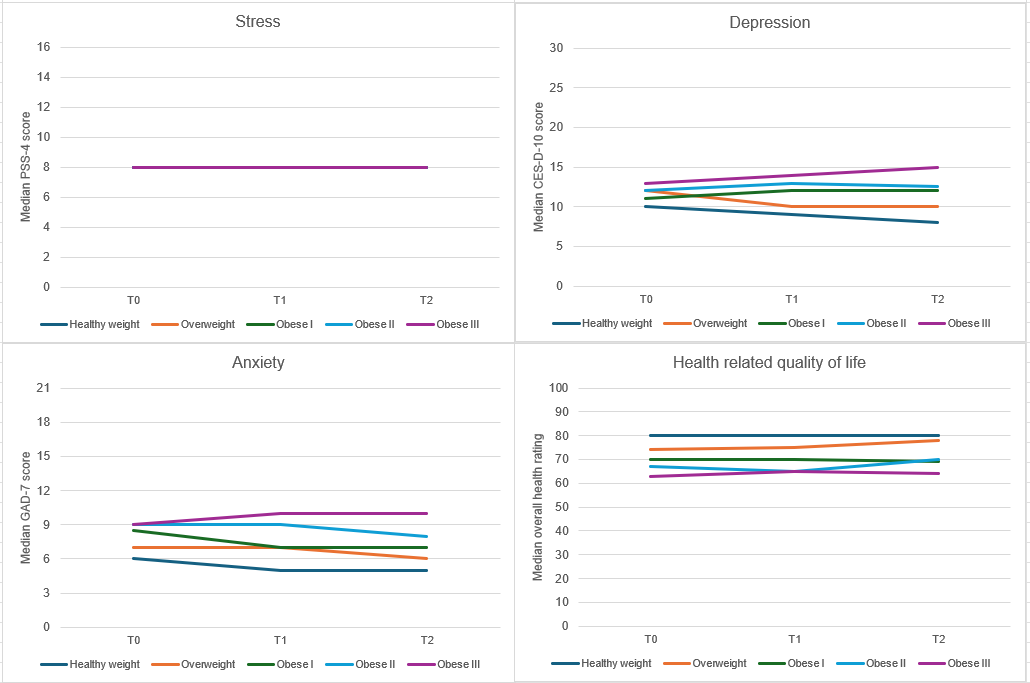


**Supplementary Figure 2.** Eating behaviour and mental health characteristics of the YOUTH cohort study participants by BMI category and timepoint

**Supplementary Table 1.** Summary of RI-CLPM outcomes by gender: unstandardised cross-lagged path regression coefficients and long run path parameters (standard error) and statistical significance of the model with gender differences for the YOUTH Cohort Study (n=501)

| **Weight & x** | **Unstandardised regression coefficients or long run path parameters (times T1 to T3)^a^** | | | | **Significance of gender models** | |
| --- | --- | --- | --- | --- | --- | --- |
|  | **x at T1 to weight at T2** | **x at T1 to weight at T3** | **weight at T1 to x at T2** | **weight at T1 to x at T3** | **χ^2^(df=4)** | **p** |
| *Weight & mYFAS 2.0* | | | | | 3.856 | 0.43 |
| Females | 0.836 (0.349) | 0.977 (0.377) | 0.085 (0.042) | 0.099 (0.041) |  | |
| Males | 0.631 (0.447) | 0.708 (0.467) | 0.025 (0.027) | 0.028 (0.029) |  |  |
| *Weight & EDEQ Global* | | | | | 6.015 | 0.20 |
| Females | 2.109 (1.300) | 1.553 (0.735) | 0.031 (0.012) | 0.023 (0.009) |  | |
| Males | -0.364 (1.006) | -0.427 (1.178) | 0.005 (0.008) | 0.006 (0.009) |  |  |
| *Weight & EDEQ Restraint* | | | | | 2.763 | 0.60 |
| Females | 1.039 (0.864) | 0.485 (0.311) | 0.042 (0.032) | 0.020 (0.011) |  | |
| Males | -0.861 (0.842) | -0.860 (0.826) | -0.016 (0.019) | -0.016 (0.019) |  |  |
| *Weight & EDEQ Eating concern* | | | | | 1.620 | 0.80 |
| Females | 1.845 (2.323) | 2.265 (2.808) | 0.032 (0.043) | 0.040 (0.053) |  | |
| Males | -0.095 (1.004) | -0.107 (1.131) | 0.007 (0.008) | 0.008 (0.009) |  |  |
| *Weight & EDEQ Shape concern* | | | | | 5.392 | 0.25 |
| Females | -0.893 (0.572) | -1.433 (0.938) | -0.002 (0.008) | -0.004 (0.013) |  | |
| Males | 0.716 (0.763) | 0.971 (1.014) | 0.010 (0.012) | 0.013 (0.015) |  |  |
| *Weight & EDEQ Weight concern* | | | | | 3.203 | 0.53 |
| Females | -0.411 (0.631) | -0.505 (0.774) | 0.002 (0.012) | 0.003 (0.015) |  | |
| Males | -0.097 (0.667) | -0.143 (0.980) | 0.006 (0.008) | 0.008 (0.012) |  |  |
| *Weight & PNEES negative* | | | | | 1.630 | 0.80 |
| Females | 0.024 (0.148) | 0.021 (0.135) | -0.060 (0.251) | -0.054 (0.223) |  | |
| Males | -0.058 (0.094) | -0.048 (0.075) | 0.018 (0.109) | 0.015 (0.089) |  |  |
| *Weight & PNEES positive* | | | | | 3.241 | 0.52 |
| Females | -0.032 (0.106) | -0.041 (0.133) | -0.060 (0.066) | -0.076 (0.076) |  | |
| Males | 0.041 (0.162) | 0.046 (0.181) | -0.006 (0.069) | -0.006 (0.078) |  |  |
| *Weight & Stress* | | | | | 3.254 | 0.52 |
| Females | 0.232 (0.240) | 0.256 (0.265) | 0.048 (0.037) | 0.053 (0.041) |  | |
| Males | 0.413 (0.428) | 0.396 (0.402) | 0.011 (0.025) | 0.011 (0.024) |  |  |
| *Weight & Anxiety* | | | | | 5.122 | 0.28 |
| Females | 0.518 (0.231) | 0.432 (0.167) | 0.191 (0.063) | 0.160 (0.053) |  | |
| Males | 0.229 (0.219) | 0.181 (0.178) | 0.072 (0.065) | 0.057 (0.051) |  |  |
| *Weight & Depression* | | | | | 3.964 | 0.41 |
| Females | 0.065 (0.166) | 0.076 (0.191) | 0.079 (0.077) | 0.092 (0.082) |  | |
| Males | 0.460 (0.224) | 0.508 (0.202) | 0.078 (0.068) | 0.087 (0.064) |  |  |
| *Weight & QOL* | | | | | 3.870 | 0.42 |
| Females | 0.049 (0.044) | 0.057 (0.053) | -0.012 (0.288) | -0.014 (0.339) |  | |
| Males | -0.065 (0.061) | -0.078 (0.059) | -0.424 (0.404) | -0.508 (0.364) |  |  |

^a^y=weight, x=explanatory variable as listed in the first column, the index is time period, 1, 2 or 3. The weight variable used in analyses was weight in kilograms.

**Supplementary Table 2.** Model fit indices for the RI-CLPM by gender

|  | **CFI** | **RMSEA** | **SRMR** | **TLI** |
| --- | --- | --- | --- | --- |
| Weight & mYFAS 2.0 | 1.000 | <0.001 | 0.020 | 1.010 |
| Weight & EDEQ Global | 1.000 | <0.001 | 0.013 | 1.007 |
| Weight & EDEQ Restraint | 1.000 | <0.001 | 0.014 | 1.012 |
| Weight & EDEQ Eating concern | 1.000 | <0.001 | 0.024 | 1.002 |
| Weight & EDEQ Shape concern | 1.000 | <0.001 | 0.017 | 1.002 |
| Weight & EDEQ Weight concern | 1.000 | <0.001 | 0.021 | 1.002 |
| Weight & PNEES negative | 1.000 | 0.020 | 0.022 | 0.999 |
| Weight & PNEES positive | 1.000 | <0.001 | 0.022 | 1.000 |
| Weight & Stress | 1.000 | <0.001 | 0.030 | 1.008 |
| Weight & Anxiety | 1.000 | <0.001 | 0.025 | 1.006 |
| Weight & Depression | 1.000 | <0.001 | 0.019 | 1.007 |
| Weight & QOL | 1.000 | <0.001 | 0.022 | 1.007 |

CFI = comparative fit index. RMSEA = root mean square error of approximation. SRMR = Standardized Root Mean Square Residual (SRMR). TLI = Tucker-Lewis Index.
